# Difference between perceptions of preceptors and newly graduated nurses regarding delay in professional growth: a thematic analysis

**DOI:** 10.1186/s12909-022-03617-y

**Published:** 2022-07-21

**Authors:** Chihiro Kawakami, Rintaro Imafuku, Takuya Saiki

**Affiliations:** Medical Education Development Center, Gifu University, 1-1Yanagido, Gifu-city, Gifu-prefecture Japan

**Keywords:** Nursing practice, Delay in growth, Preceptor, Difference of perceptions, Preceptee, Nursing education

## Abstract

**Background:**

Smooth reciprocal relationships enable a preceptee’s growth, and it has been suggested that without such relationships, the preceptee may not be able to grow successfully. This study explored the differences in perceptions by matching the perspectives of both the preceptees who did not make progress in workplace adjustment and their preceptors. Identifying the differences in perceptions between the two groups is important for improving nursing education and the relationship between preceptees and preceptors.

**Methods:**

A pair of nurses who had been with the company for less than 3 years and who had previously been transferred or had resigned due to poor workplace adjustment were designated as preceptees, and those who had directly supervised the preceptee during their first year of employment were included as preceptors in the study. A 50-minute semi-structured interview was conducted separately to examine the perceptions of the preceptee and preceptor. A thematic analysis was used to analyse the interview data.

**Results:**

This study explored the differences in perceptions regarding the clinical practice of nursing between preceptors and their preceptees who did not make progress in workplace adjustment during nursing education; six themes were identified. After interviewing both sides, it became clear that the same event was interpreted differently depending on their positions, perspectives, and contexts. As the preceptees were nurses who had left or had been transferred, the existence of these differences in perceptions suggests that these factors may impact their departure or transfer. However, we do not aim to place blame on one side or the other for the preceptee’s turnover or transfer and would like to consider effective support, not only for the preceptee, but also for the preceptor.

**Conclusions:**

It is necessary to examine nursing education on the premise that differences may occur depending on the position and role of nurses in the workplace and to look at curricular framework changes to bring in a systemic influence towards the training of young nurses.

## Background

Training newly recruited medical personnel is an important objective in medical education. In the medical field, it is essential to secure and train reliable nurses to provide appropriate treatment and care for patients. In the field of nursing, preventing the early turnover of nurses within the first year of employment is an urgent issue [1, 2]. However, in 2014, WHO and World Bank calculated a global shortage of nine million nurses and midwives [3]. Furthermore, currently in Japan, about 10% of new nurses leave the workforce early—within 1 year of starting work [4, 5].

In general, the reasons nurses leave their jobs include organisational factors, such as the work environment and organisational culture, and personal factors, such as job satisfaction and burnout; these factors are complex and intertwined [6]. Factors related to the preceptor turnover (hereinafter referred to as “preceptor” for nurses who are educators) include psychological burden [7], problems related to nursing practice ability [8], reality shock [9] after joining the company, and organisational factors such as human relations and work environment [10, 11]. In order to prevent such early turnover, the presence of a preceptor who supports the personal needs of newly hired nurses (hereinafter referred to as “preceptee” for new and young nurses) in the workplace and develops their nursing practice skills is vital. However, in reality, there are many preceptors who have difficulty dealing with preceptees [12, 13]. Preceptors find it difficult to comprehend the level of understanding of the preceptees and to communicate smoothly according to the situation [14]. Thus, providing individualised guidance to new nurses is difficult [15].

To develop effective nursing education, it is important to examine the relationship-building process and perceptions of practice between preceptors and preceptees from their perspectives. This is particularly important for postgraduate nursing education because, while nursing students as preceptors before graduation can have a university faculty member who coordinates their relationship with their preceptor, after graduation, this changes to a one-on-one relationship. However, studies from such a comprehensive perspective are limited. In extant studies, when the preceptor and the preceptee were surveyed about their satisfaction with the preceptorship, their job satisfaction was high and the preceptor’s strength was reflected in the preceptee’s ability to fit in with the new unit, teach, and share knowledge. Conversely, the preceptee indicated interpersonal relationships, communication, and professional development as strengths of the preceptor and teaching, collaboration, and critical care as weaknesses [16]. They also stated that it is important for the personal characteristics and learning styles of both parties to match [16]. In one study that explored the perceptions, experiences, and needs of both preceptees and preceptors regarding preceptorship, it was found that the social role of the preceptor, providing autonomy to preceptees, communication and the use of technology, involvement of nursing managers, and learning styles were the most important factors [17]. Further, it was noted that the relationship between the preceptor and the preceptee was important, and when the preceptor was relationship-oriented, the preceptee’s independence was hindered and when the preceptor was task-achievement-oriented, the relationship was tense and the feedback was critical [17]. Smooth reciprocal relationships enable the preceptee’s growth, and it has been suggested that without such relationships, the preceptee may not be able to grow successfully.

This study explored the differences in perceptions by matching the perspectives of both the preceptees who did not make progress in workplace adjustment and their preceptors. Identifying the differences in perceptions between these two groups is important for improving nursing education and the relationship between preceptees and preceptors.

## Methods

### Study design

This study was designed based on an exploratory case study approach, which aims to gain an extensive and in-depth description of a complex phenomenon that has not been fully studied. Yin [18] defines a case study as ‘an empirical inquiry that investigates a contemporary phenomenon within its real-life context’. Being informed by the interpretivist paradigm [19], this study explored how and what perceptual differences between preceptees and preceptors occurred through social interactions in the processes of workplace adjustment. To enhance the methodological rigor and report writing, this study followed the consolidated criteria for reporting qualitative research (COREQ) which offers a 32-item checklist for interviews and focus groups [19] .

### Participants

The pairs recruited in this study comprised those individuals who had newly graduated and had left the hospital within the first 3 years due to workplace transfers or workplace maladjustment (preceptees). Furthermore, the preceptors who had directly supervised them during their first year were also included.

We recruited participants with the cooperation of the Department of Nursing at two teaching hospitals with 600 or more beds. To be eligible for participation, nurses had to be qualified but exhibit a delay in professional growth for the first 3 years. In most hospitals in Japan, the Department of Nursing utilises a three-year training programme following the employment of newly graduated nurses as a means for developing nurses. To determine whether a nurse is competent, both teaching hospitals employ the training guidelines of the Japanese Nursing Association’s (JNA) Clinical Ladder [20]. This guideline consists of training of new nurses, development of mentors for new nurses, training plans, and nurse evaluation based on the clinical ladder established in Dreyfus model [21]. In this study, we defined delayed professional growth as the nurse not meeting the standards outlined in the JNA Clinical Ladder training guidelines within 3 years of employment, as judged by their instructors and nurse directors, resulting in their resignation or transfer to another division. Here, we termed nurses with delayed professional growth as preceptees. The person who was in charge of educating that preceptee in the first year was designated as the preceptor, and the pair was targeted. Preceptors are nurses who have met the criteria of the Clinical Ladder (Level III, approximately 5 years or more after entering the profession) as indicated by the JNA and are appointed by the director of the nursing department of their hospital [20]. They then undergo, in total, approximately 1 week of teaching and learning support training, usually throughout the year, at their hospital or at the prefectural nursing association.

After obtaining informed consent verbally, the Department of Nursing scheduled interviews with each participant. The author (the interviewer) was introduced to the participants on the day of the interview. The author explained the study objectives prior to obtaining the respondents’ written consent in order to ascertain their will to participate in the interview.

Ten pairs of participants from the two teaching hospitals participated, comprising a total of 20 participants.

### Ethical considerations

This study was approved by the Institutional Review Board of Gifu University (Approval Number 25–385). As for the content of the preceptors’ and preceptees’ interview comments on the preceptees’ work performance, confidentiality was assured.

### Data collection

The interviews were conducted in Japanese by one lead author, who had enough experience of conducting interviews. Interviews were conducted from March to August 2014. A 50-minute semi-structured interview was conducted separately to examine the perceptions of the preceptee and preceptor. All interviewees were interviewed individually in a private room where psychological safety and privacy were ensured so that the content of the interview would not be overheard by others. The interviews were recorded after obtaining the interviewee’s consent. In the interview, the preceptors were asked about memorable events in their corresponding preceptee’s nursing clinical practice (work). The preceptees were also asked about memorable events in their nursing clinical practice (work). The interview guide was developed by the authors according to prior literature as follows [14–17]. The interview guide was as follows:

#### Preceptee

Looking back on your clinical nursing practice (work) in your first year of employment, is there anything that left a strong impression on you?

- Please tell us specifically about this event.
- What do you think were the positives/negatives (cause) of the event?
- How did you feel about the incident?’

#### Preceptor

Are there any memorable events in the preceptee’s nursing clinical practice (work)?

- Please tell us specifically what happened.
- What do you think were the positives/negatives (cause) of the event?
- How did you feel about the incident?’

### Data analysis

A thematic analysis with an inductive approach was used to analyse the interview data [22, 23]. The recorded data was contracted to a professional transcription company, and its content was verified by the author. Two researchers (CK, TS) independently read the transcripts several times to understand the text, found the keywords in the text, coded the data from the keywords, and finally, organised these codes into themes by repeatedly discussing the initial theme to modify and integrate the final theme. Step-by-step coding was done with reference to the literature [22, 23]. Co-authors, whose areas of expertise were nursing and health professions education reviewed the data from a variety of perspectives. The validity of the analysis was verified by another researcher (RI), whose area of expertise was education, through a discussion of the final themes until a consensus was reached.

## Results

### Participants

Through the nursing departments of the two university-affiliated hospitals, the researcher asked the ward managers to name the target pairs, obtained the informal consent of both members of a pair, explained the results to them, and obtained their written consent. After all authors discussed the data gathered, it was confirmed that the data was saturated and no more new data would be generated, bringing the total number of participants to 20. Consequently, 10 pairs consented to the study. The demographic data of the participants were as follows: ten preceptors were female. The ages of the preceptors at the time they precepted ranged from 26 to 35 years (mean 28.7 years), and at the time they were interviewed ranged from 28 to 38 years (mean 30.6 years). Eight preceptees were female and two were male. They were between 23 to 25 years old at the time of precepting (mean 23.4) and between 24 to 27 years old at the time of interviewing (mean 25). The status of each pair is described below (Table 1).

**Table 1:** The status of preceptees and preceptors

pair	Preceptee’s situation and experience	Education experience as a preceptor
A	Second year working as a nurse, transferred from another hospital, planning to retire.	Second time in charge of a rookie.
B	Second year on the job, department transfer in the middle of the first year, scheduled to retire.	Second time in charge of a rookie.
C	Third year as a nurse, transferred to a different department in the middle of the first year, considering retirement, seeing a psychiatrist.	For the first time.
D	5 years on the job, transferred to a different department in the middle of the first year and continued to work there, saw a psychiatrist.	Second time in charge of a rookie.
E	Second year of service.	For the first time.
F	Third year of service.	Multiple rookie assignments.
G	Retired after 2 years of service and will be transferred to another hospital.	Multiple rookie assignments.
H	Retired after 3 years of service and will be transferred to another hospital.	For the first time.
I	Two years on the job, with psychiatric consultation.	For the first time.
J	Two years on the job, seeing a psychosomatic doctor.	For the first time.

### Difference in perception between the preceptee and the preceptor

From the interviews, the following six themes were extracted about the perceived differences between preceptees and preceptors regarding the former’s clinical practice. Sub-themes and codes are also tabulated together (Table 2).

**Table 2:** Themes, sub-themes, and codes

	Theme	Sub-Theme	Code
1	Preceptee’s ability to carry out their work	(1) Perceived differences in manual dexterity when performing tasks (2) Differences in perceptions of time allocation and pace of completing tasks (3) Differences in perceptions of procedures for learning	Preceptor ・being bad at dealing with things ・clumsy ・very slowly ・the work learned is reset the next day ・different pace from mine Preceptee ・work overload ・very time consuming ・too fast for me to keep up ・perception of being able to
2	How to direct preceptee’s awareness (attention) during work	(1) Differences in perception of focused concentration (2) Differences in perceptions of handling urgent situations	Preceptor ・lack of awareness of the whole ・overconcentration on small things ・overreaction to other things Preceptee ・perception of being too busy ・sense of responsibility
3	The ability of the preceptee to apply knowledge		Preceptor ・can learn from textbooks, but cannot apply that learning to clinical practice ・clinical experience is not utilised Preceptee ・learned knowledge from textbooks ・don’t know how to apply this to clinical practice
4	Self-evaluation ability of preceptee	(1) Differences in evaluations of my growth as a learner (satisfied with my growth) (2) Differences in the evaluation of actual work performance (low evaluation standards for new nurses)	Preceptor ・not up to standards ・high self-esteem Preceptee ・satisfied with growth ・highly evaluates own performance
5	The nature of communication in the learner–instructor relationship	(1) Differences in attitudes toward learning in learner–instructor relationships (2) Differences in perceptions of how to check operations	Preceptor ・response that shows no respect ・reaction different from prediction Preceptee ・concerned about the preceptor’s vague answers ・not aware of any communication problems
6	Conducting business in a collaborative manner	(1) Differences in perceptions of the significance of asking for help from the team (2) Differences in interpretation of team collaboration	Preceptor ・not understanding the significance of asking for help ・not understanding in the others situation ・differences in how responsibility is perceived Preceptee ・must be worked alone ・someone else will do it

Next, we summarise the definitions by themes and the descriptions of the preceptee and preceptor pairs. The following table summarises characteristics of the pairs that showed cognitive differences in Themes 1 to 6 across both interviews (Table 3).

**Table 3:** Emergence of a theme for each pair

pair	Theme 1	Theme 2	Theme 3	Theme 4	Theme 5	Theme 6
A	✓			✓	✓	✓
B			✓			✓
C	✓			✓	✓	
D	✓				✓	
E	✓		✓	✓	✓	
F	✓	✓	✓		✓	
G	✓			✓	✓	
H	✓	✓		✓	✓	✓
I			✓	✓	✓	✓
J	✓		✓		✓	✓

### Theme 1: Preceptee’s ability to carry out their work

#### Definition of theme 1

Performing nursing clinical work in a methodical manner is important, and both parties recognised that this requires hand dexterity, manual dexterity, and the ability to learn to complete the work in a timely manner. Generally speaking, with practice and experience, a person can gradually improve their ability to perform the job and complete it in less time. However, both parties understand that an inexperienced and unaccustomed preceptee’s job performance may neither be adequate nor timely; compared to the nurses that the preceptor has taught in the past, this preceptee might be taking longer than is acceptable.

##### Sub-theme (1): Perceived differences in manual dexterity when performing tasks

Both members of a pair were aware of the preceptee’s manual clumsiness. However, the preceptee had a hard time accepting this lack of manual dexterity, exhibited a strong desire to improve, and often felt impatient, saying ‘I can do it’ when unable to do something.

In contrast, the preceptor felt that the preceptee is too clumsy and takes more time than allocated; this feeling can be regarded as dismay. In addition, the preceptor judged the workflow as ‘disorganised’ and ‘not memorised.’

Pair E

Preceptee

‘When I was doing detailed work, I was awkward or not to the point, or maybe I am not good at detailed manual skills (I think). I thought it was natural that my level would increase after the first year, but I could not do it. I could not admit that I could not do it because I saw my peers who were doing so well. I did not want to admit that I could not do it. I was so impatient that I did not want to drop out, so I said that I will do it even when I could not do it. I thought I was being too tall for my own good.’

Preceptor

‘She/he was very clumsy with her/his hands; for example, a pubic wash would take 40–50 minutes. She/he was so nervous and sweaty that she/he could not put the gloves properly, and the items were not properly positioned—the patient kicked and spilled them; she/he could not connect the instruments properly for the intravenous drip or the blood collection. It was like she/he could not do it smoothly and it was a mess. In the end, I think she/he did not remember the procedures perfectly, and in April (right after she/he joined), she/he already had a gap with her peers, which we both knew and were worried about.’

##### Sub-theme (2): Differences in perceptions of time allocation and pace of completing tasks

Both members of a pair were aware that it takes the preceptee a very long time to complete the day’s work. The preceptee was aware that this was due to the fact that there were many tasks. In addition, the preceptee was underinformed about the work and in a state of anxiety when performing it. In contrast, the preceptor worked as usual, but was concerned that the preceptee was not able to match this pace. The preceptor felt that the preceptee’s work was slower than the required pace.

Pair D

Preceptee

‘The routine was too busy for me to keep up with, and I had to do my daily work. It was common to work overtime until 8 or 9 pm. I wish I had been taught nursing skills hands-on. Once I had learned how to perform a bed bath; I was forced to do it alone. I was never given a checklist to confirm my skills, and if I said I could do it, I was forced to do so alone.’

Preceptor

‘It was the first day of working with her/him. I walked with the her/him to the patient room, but when I turned around, she/he was not following me. She/he and I did not have the same flow of time or did not match or (she/he) was very slow, and I wondered if it was okay. She/he is quiet and does not talk unless I talk to her/him, so I was a little confused about dealing with someone with that personality. When I thought that I could do things at this pace, or that other new nurses could do things at this pace, I compared myself to her/him, recognising that she/he was slow. It takes me a long time to do each thing with her/him, and I think I am not quite to the point.’

##### Sub-theme (3): Differences in perceptions of procedures for learning

Both members of a pair were aware that the preceptee was working hard to learn the job. However, the preceptee did not think that the learning was time consuming and presumed, on the contrary, that trying to find a way to remember things in an organised way is not an advisable way to learn. In contrast, the preceptor recognised that the preceptee’s learning method was not appropriate, and that concepts, unless stored in an organised manner, cannot be retrieved from memory.

Pair A

Preceptee

‘I told one staff member that it took me about five hours to learn, and she/he asked, “Why does it take you five hours to do that?” When I told her/him, “This will take you about 20 minutes to learn”, she/he said, “That takes five minutes, doesn’t it?” She/He said, “No, I do not think so.” I was trying my best to remember, but it seemed to take me four times longer than others. I used to think that being to the point was not a very good thing, but I thought that is what you need to do to get a lot done.’

Preceptor

‘I have a marker line through all of my disease standards, and I have heard that she/he colour-codes them as, but this makes me wonder if she/he knows what is important. Even though I told her/him to summarise the important points by handwriting, I often wondered if this was the point of view to summarise. It is understandable that she/he did not know because it was her/his first time, but she/he did not remember what I taught, and she/he did not remember if she/he wrote it down or not. If she/he had considered it important and written it down, she/he would remember it, but she/he did not, or perhaps, she/he could not recall it from of memory. I was patient with her/him at first, but then I got frustrated, and then I just gave up.’

### Theme 2: How to direct preceptee’s awareness (attention) during work

#### Definition of theme 2

It is important to maintain a broad perspective in nursing clinical work, such as planning ahead for the next step and observing the overall situation. The preceptee was motivated to perform his/her work and role. Conversely, the preceptor’s evaluation stated that the preceptee was distracted, neglected important perspectives, and was unable to fulfil his/her role.

##### Sub-theme (1): Differences in perception of focused concentration

Both members of a pair recognised that it was important to perform nursing tasks while taking a broad perspective and responding to situations. However, they were aware that the preceptee was not able to do so; the preceptee was overloaded with work and oblivious to her/his environment, and did not recognise that she/he forgot something when doing something else quickly. The preceptor judged that the reasons for this were the preceptee’s inability to focus on anything else besides the present task and excessive focus only on the execution of his/her work, ensuring that other things do not come into view.

Pair H

Preceptee

‘I have multiple assignments where I do not do more than one care task at a time, and I am not good at that. I have never worked in an overcrowded schedule before, and I did not expect this job to be that busy. If I try to rush things, I fail, so I cannot do things quickly. But if you do not do it quickly, you will not finish. When I would be thinking about doing something and something else would come in, I would forget what I was trying to do initially; later, the staff would tell me that I had not done it, and then I would finally remember.’

Preceptor

‘I was very shocked when a post-op patient was complaining of pain right in front of she/he, and she/he were asking the patient if she/he was numb, and they did not think of doing something about the patient’s pain. Normally, I would try to do something about the patients’ pain. However, they are thinking about other things, or can only do one thing at a time. When I ask, “What did the patient just say to you?” or if I ask, “What did you say?” they say, “I am going to go back and ask again.”’

##### Sub-theme (2): Differences in perceptions of handling urgent situations

Both sides recognised that the preceptee was trying to respond to urgent situations as quickly as possible. The preceptee felt that it was her/his ‘role and responsibility as a newcomer’ to respond to urgent tasks and she/he tried to do so. However, the preceptor did not expect the preceptee to respond in such a way and instead, expected the preceptee to first develop the responsibility of completing her/his own work.

Pair F

Preceptee

‘When I am in charge of a patient who needs a lot of work, I wish there was another person to help. I feel like I have to do this and that while I am doing my duties. When I have my own work to do, and the nurse call rings and says, “It is someone from B team”, I feel like I have to go. When I heard a senior nurse in the staff room say, “Newcomers do not see many patients, so you should at least take nurse calls”, I knew I had to. In my head, I know I do not have the time to do that. It is not my patient, and I do not really want to go, but I think the new guy has to go.’

Preceptor

‘When I ask someone to run an errand, she/he takes the initiative but the rest of the staff is worried if she/he can manage it. I try to talk to her/him on the assumption that she/he has finished her/his work, but I have to check first. I need to be sure. I can do what I say, and I can order the care and duties, but if there is a sudden event or I help someone else, I forget about myself. I am often so focused on what I am doing that I do not do my own things. She/he does not mind being asked to do things, so she/he takes the initiative, but when that happens, she/he inevitably neglects her own tasks.’

### Theme 3: The ability of the preceptee to apply knowledge

#### Definition of theme 3

In clinical nursing practice, it is important not only to recall and understand what has been learned, but also to think in terms of application from learning to practice. The preceptee and preceptor both recognised that knowledge at the level of recall using a textbook was not an issue for the preceptee. However, the preceptee did not know how to apply that knowledge to practice and was instead focused on increasing the hours of study. In contrast, the preceptor felt that the preceptee’s knowledge was limited to the textbook and not connected to the patient’s clinical condition.

Pair F

Preceptee

‘Studying is not my weakness; I can do it if I try, but I often cannot use it (factual knowledge) in patient assessment and practice. When asked by the preceptor, “What is your reasoning?” I can tell that the patient has a symptom, but I cannot make a care plan.’

Preceptor

‘I think she/he is fine and able to do. I can sit in front of the computer (nursing record) and say, “What about this observation item?” or “Is this associated factor correct?” I would say, “Oh, this is it. No, it is not. I think you know what I am talking about. But I cannot apply it to practice, and I often cannot do it with my patients.”’

Pair B

Preceptee

‘I study because I think I am not good enough, but I cannot apply the knowledge in my head to what I am doing. Even if I study, I cannot make use of it; it is like a spiral. I try my best, but there are patients from many different departments (in the ward); so, no matter how much I study every day, I cannot keep up. I do not feel like I am nursing, but rather just doing what I am told on the spot. (The preceptor) told me that what I am doing and what I am studying are not connected and I understand that, but I do not know how to connect them.’

Preceptor

‘I encouraged her/him to look back at today’s patient and draw a related diagram, but I got back a diagram that was so far removed from the current problem that it looked like something from a textbook. The report was quoted directly from the textbook and copied verbatim.’

### Theme 4: Self-evaluation ability of preceptee

#### Definition of theme 4

In nursing clinical practice, the ability to accurately self-evaluate is important for advancing and deepening learning. In the review and evaluation of the preceptee’s practice to date, the preceptee stated that there were no problems in learning and growth. In contrast, the preceptor was concerned that the preceptee had not sufficiently mastered the skills to be able to function independently.

##### Sub-theme (1): Differences in evaluations of my growth as a learner (satisfied with my growth)

Both parties have been working very hard in their nursing clinical practice. The preceptee felt they had mastered nursing skills through practice, did not feel clumsy, and had a relatively high self-evaluation. However, the preceptor’s evaluation of the preceptee’s skills was low; the preceptor continuously monitored the preceptee due to unsurety about the preceptee performing nursing skills without supervision.

Pair H

Preceptee

‘I think I was able to take blood samples after doing it a few times, rather than being taught. As for the finer techniques, that is how I got better at most of it. The manual skills were not that much of a problem.’

Preceptor

‘She/he had mastered very few nursing skills and I was worried about letting her/him work independently because sometimes she/he could use the intravenous drip and infusion pump, but sometimes she/he could not. There were times when I wondered why she/he could not do something today that she/he could yesterday. The blood collection was almost independent in the second half (of the year), but I was continuously supervising because there was always something that would be left out.’

##### Sub-theme (2): Differences in the evaluation of actual work performance (low evaluation standards for new nurses)

We recognised that both parties had been working very hard in their nursing clinical practice. The preceptee was unaffected by the fact that she/he was being properly taught nursing skills and felt that she/he was growing. The preceptor, in contrast, perceived no growth in the preceptee’s nursing skills and was embarrassed by the staff’s ridicule of slow growth.

Pair G

Preceptee

‘I was taught the techniques whenever required from time to time, and I did not have that much trouble because of my training, so I did not have any difficulty with them. I think I adequately managed the procedures.’

Preceptor

‘I was asked to take the same patient the next day because I could do it yesterday. However, it was reset, or rather, there were times when I forgot the next day even though the skill was done the day before. When no signs of progress (in nursing skills) were shown, the people around me started to wonder, “How are you going to teach them?”’

### Theme 5: The nature of communication in the learner–instructor relationship

#### Definition of theme 5

Communication (reporting, communication, and consultation) with other staff members is important when there is uncertainty about how to perform a task. The preceptee did not feel that there was a problem with her/his way of communicating. The preceptor, however, felt that the preceptee’s way of communicating as a learner was not satisfactory.

##### Sub-theme (1): Differences in attitudes toward learning in learner–instructor relationships

The preceptee never had any problems with interpersonal relationships. However, preceptors felt uncomfortable that the preceptee did not exhibit a learner-appropriate attitude and pretended to understand even though that was not the case.

Pair I

Preceptee

‘I thought I had no problems with it (communication). I do not remember ever having that much trouble with interpersonal relationships either.’

Preceptor

‘When I am talking to her/him, her/his reaction is always different than what I expect from a preceptee. When I provided advice in an educational capacity, the other new nurses would respond “yes”, she/he would respond “yes, I knew that.” So, I thought she/he said so because she/he was advised something she/he knew, but she/he did not know.’

##### Sub- Theme (2): Differences in perceptions of how to check operations

The preceptees paid attention to the preceptor to know when to report work and how to react. In addition, they found it difficult to respond to a preceptor’s inconsistent responses.

However, while the preceptor acknowledged the preceptee’s hard work and desperation, the preceptor perceived that important information was not communicated. The preceptor did not know how to respond to the preceptee, and the responses became more withdrawn.

Pair J

Preceptee

‘If I say, “I am going to go do this”, and you answer, “Well, I will do some other work while you doing that”, it would go relatively smoothly. I do not know whether you can hear me or whether I am getting through to you while I am looking at the (computer) screen, but I am not sure whether I should say it again. I thought the preceptor was concentrating, so when I said it again, she/he sometimes would reply, “I heard you earlier.” Another time (in the middle) I did not get a response, but I tried to go with the idea that she/he heard me (because of the last time), and she/he said, “If I did not respond, then she/he did not hear me, so it is like (you) did not say it.”’

Preceptor

‘She/he greets me well and asks me anything freely, but sometimes important things are left out; so, I feel like, “What are you saying every single time?” I can tell that they are desperate and want to do their best. However, since they are missing something important, they are spinning their wheels, they cannot do even the most trivial duplication of work, and they do not know what to do first. I do not know what to do first, and tend to be hard on them.’

### Theme 6: Conducting business in a collaborative manner

#### Definition of theme 6

In the same ward, it is important to perform tasks in a collaborative manner. However, the preceptee was self-directed and had little awareness of working with the team and supervisor. Conversely, the preceptor was a member of the team and had a high sense of collaboration, and this was also expected of the preceptee.

##### Sub-theme (1): Differences in perceptions of the significance of asking for help from the team

The preceptee believed that when a problem arose, she/he should solve it without asking for help. At the root of this was a desire to not be considered inferior. The preceptor, however, perceived that the preceptee’s inability to seek help was due to a lack of understanding of the impact on patients and their needs.

Pair G

Preceptee

‘I thought I had to (figure it out myself), so I thought I could not just say, “I do not know”, or “I have never done it before.” I mean, I did not think of asking. The preceptor said to me, “If you have never done it before, you have to tell me, right? Do you understand?” I said, “I don’t know”, and preceptor said, “You have to tell me what you do not know, don’t you? I do not know. However, you are still at this level”. I could not say it because I was afraid of what would happen if the preceptor thought that I was still at this level.’

Preceptor

‘I told her/him to call someone on the nurse’s call when the patient got up from the wheelchair on the bedside table, but I do not know if everyone was too busy to call, or if she/he did not call, but she/he started to help the patient herself. The patient had a deteriorated cognitive condition, and the chair was placed in a halfway position. So, I told her/him, “That kind of thing is scary”, and she/he cried a lot, so I thought that she/he understood how scary it was (to assist alone) and the need to report it.’

##### Sub-theme (2): Differences in interpretation of team collaboration

The preceptee was unappreciative of other staff members shouldering the work she/he was unable to do. Conversely, the preceptor coordinated the work behind the scenes so that the preceptee was not overburdened. However, she/he felt pressure from her/his staff, which resulted in more work for her/him, and she/he felt that she/he was burdening her/his staff with more work.

Pair A

Preceptee

‘I think the pair probably did a lot of work, but they did a lot of things that I did not do, and I was thought, “Oh, it was over before I knew it”’

Preceptor

‘She/he was taking so much time to get basic information about the patients. She/he would drastically reduce her/his duties or fix the duties and tell her/him to focus only on that. I told them to return to the station at 4:00 (in the evening) so that there would be no overtime. It might have been difficult for the staff around me because they had to take on that much work. So, I felt that (the other staff) had an atmosphere of not wanting to be under my guidance.’

## Discussion

This study explored the differences in perceptions regarding the clinical practice of nursing both the preceptees who did not make progress in workplace adjustment and their preceptors during nursing education; six themes were identified.

After interviewing both sides, it became clear that the same event was interpreted differently depending on their positions, perspectives, and contexts. As the preceptees were nurses who had left or had been transferred, the existence of these differences in perceptions suggests that these factors may have an impact on their departure or transfer. However, we do not aim to place blame on one side or the other for the preceptee’s turnover or transfer and would like to consider effective support, not only for the preceptee, but also for the preceptor.

### From the perspective of preceptors

The preceptor tended to ascribe the cause of the preceptee’s poor performance to the preceptee alone. In other words, the preceptor tended to attribute the preceptee’s inability to perform to internal factors such as personality traits, ability, and effort—a fundamental attribution error [24, 25]. Furthermore, the educational repertoire did not match the individuality of the preceptee. For example, if there was no growth in the preceptee, Theme 1, Sub-theme (1), preceptors attributed preceptees’ inability to clumsiness, or to their nature or personality; Theme 1, Sub-theme (2), preceptors tended to use ‘self’ or ‘nurses in charge of education in the past’ as the standard and then rated preceptees as not able; Theme 6, Sub-theme (3), measures to reduce the workload of preceptees have been attempted, but it is hard to say find an effective way to educate preceptees.

In a survey conducted by Gregg et al. [15], the most common difficulty for new nurse educators in fulfilling their role in nursing education was ‘teaching to meet individual needs’. The preceptors in this study did not give due consideration to the individuality of the preceptee and erred in addressing the most significant area of development. Therefore, it is necessary to prepare several educational methods and scaffolds that can be adapted to the individuality and diversity of preceptees, especially nurses who grow slowly, and to train preceptors and educators in these methods.

The preceptor was disappointed with the preceptee’s poor performance and negative feelings were associated with it. This suggests that preceptors and preceptees influence each other’s relationship. The preceptor gives negative evaluations to the preceptee because the preceptee ‘cannot do’ more than she/he imagined, and this also causes negative emotions. For example, Theme 4, Sub-theme (2), feeling uneasy about preceptee. Furthermore Theme 1, Sub-theme (3), Theme 5, Sub-theme (2), the preceptor is frustrated.

The preceptor understands that the preceptee is not able to do her/his work, but experiences negative affect because she/he is not able to do it as well as imagined (mood congruent effect) [26]. The preceptor understands that the preceptee is unable to perform the task, but experiences negative affect because the preceptee is unable to perform the task as expected. As a result, the preceptor’s attitude may become harsher, and it may become difficult to maintain a good preceptor-preceptee relationship. As a result, an irreversible misalignment of the mutual relationship was identified. To prevent deterioration of the mutual relationship between preceptors and preceptees, training in emotional control (e.g. anger management) may be necessary [27].

In addition, the importance of developing a human resource environment in the workplace that is conducive to and supports the education of preceptors was clarified. Theme 4, Sub-theme (2), Theme 6, Sub-theme (2), the staff takes the position that they do not want to be involved, leaving the preceptor to educate the preceptee as if they were someone else.

Despite this, they feel that the responsibility of failure in educating the preceptee is placed solely on the preceptor. Hyrkas et al. [16] found that in the relationship between the preceptor and the preceptee, when the preceptor is in a situation where his efforts are rewarded, the preceptee’s satisfaction also increases and the preceptor’s commitment to the role is strengthened. Therefore, it is important to review the support system for preceptors and foster a culture of human resource development in the workplace.

### From the perspective of preceptees

Preceptees seemed to blame their inability to perform their own work because they were not sufficiently educated. Theme 1, Sub-theme (2), they do not check the techniques with a checklist, and once you have experienced them, you are encouraged to be independent the next time. Theme 4, Sub-theme (2), this can be seen from her statement that she/he has been able to do this in her/his nursing clinical practice rather than being taught. This is due to the self-serving bias [28, 29], in which one’s own actions are reasoned in one’s own favour, and the preceptee places the blame for her inability on external factors (attribution), namely, that she/he was not properly taught by the preceptor.

In Theme 4, it was found that the preceptee’s self-evaluation tended to be higher than the preceptor’s evaluation of work performance. Theme 6, Sub-theme (1), the preceptee had never felt the need to communicate or build interpersonal relationships, and it seemed as if this was a difficulty she/he had only faced when she/he entered the workforce.

It can be said that this preceptee experienced the Dunning-Kruger effect [30, 31], in which people with lower abilities tend to estimate their own abilities more highly. Furthermore, in Theme 1, Sub-theme (1) and Theme 6, Sub-theme (1), the preceptee is impatient and does not want to admit that he or she is not able. This is the self-evaluation maintenance motive, which is a universal basic motive for human beings to maintain their own evaluation [32]. In this self-evaluation maintenance model, in order to avoid self-evaluation, people maintain psychological distance from others who have high performance levels (in this case, the preceptor). As shown in Table 1, the preceptee may seek psychiatric treatment due to maladjustment in the workplace or personal relationships. Therefore, it is necessary for the preceptor and the preceptee to build a trusting relationship by collaboratively discussing goal setting and learning methods under an educational alliance [33]. For example, using the R2C2 model, consisting of the Relationship building, Exploring Reaction, Exploring Content, and Coating steps, may be recommended as a model that can resolve the mutual relationship through collaborative feedback and coaching [34].

### Limitations of the study and future directions

The present study has several limitations: it was difficult to ask both the preceptor and the preceptee to reflect on the same past events and situations, and therefore, we could not match them to the same situations. In addition, since the data were collected at a limited number of sites, caution should be exercised when applying the results to other contexts—the relationship between preceptees and preceptors is not easily generalised. As a question for further research, the relationship between the preceptor and their preceptee who has shown appropriate growth as a nurse should also be explored. Furthermore, as the results were obtained in the field of nursing education, they cannot be generalised to other fields. Further research is needed to explore if the same results of relationship building can be obtained in other health professions.

## Conclusion

In this study, six thematic cognitive differences were identified between preceptees and preceptors regarding the clinical nursing practice of preceptees who were not well adjusted to the workplace: the preceptee’s ability to carry out their work, how to direct preceptee’s awareness (attention) during work, the ability of the preceptee to apply knowledge, self-evaluation ability of preceptee, the nature of communication in the learner–instructor relationship, and conducting business in a collaborative manner. These six themes were found to be caused by differences in the perspectives and evaluation criteria of both parties. It is necessary to examine nursing education on the premise that such differences may occur depending on the position and role of nurses in the workplace; it is also important to consider curricular framework changes to encourage a systemic influence towards the training of young nurses.

## Data Availability

The data supporting the conclusions of this study are not publicly available in order to maintain the privacy of the participants’ educational records in the workplace, but are available from the corresponding author on reasonable request.

## Abbreviation

JNA: Japanese Nursing Association
